# Alveolar iodine tampon packing after impacted third molar surgery improves oral health–related quality of life and postoperative sequela: a randomized study

**DOI:** 10.1007/s10006-020-00898-9

**Published:** 2020-08-29

**Authors:** Jerome A. Lindeboom, Jacco G. Tuk, Patrick Möllenkamp, Arjen J. van Wijk

**Affiliations:** 1 Department of Oral and Maxillofacial Surgery, Amsterdam University Medical Center, Meibergdreef 9, 1105 AZ Amsterdam, The Netherlands; 2 Department of Oral and Maxillofacial Surgery, Amstelland Hospital, Amstelveen, The Netherlands; 3 Department of Social Dentistry, Academic Centre for Dentistry Amsterdam, Vrije Universiteit Amsterdam, Amsterdam, Netherlands

**Keywords:** Third molars, Alveolar packing, Pain, OHRQoL

## Abstract

**Objective:**

The aim of this study was to evaluate the effect of an iodine tampon on postoperative discomfort after surgical removal of a mandibular third molar.

**Material and methods:**

Patients were randomly assigned to two groups: one group received an alveolar iodine-containing tampon in the extraction socket (*N* = 44), and the other group used a disposable syringe (Monoject®) to rinse the wound (*N* = 43). Postoperative discomfort was assessed with the Oral Health Impact Profile-14 (OHIP-14) questionnaire, Pain Intensity Numerical Rating Scale (PI-NRS), and questions about self-care and discomfort.

**Results:**

This study included 87 patients (52 women and 35 men) with an average age of 26.47 years (SD, 6.36). The mean OHIP-14 sum scores were significantly lower in the iodine tampon group compared with the Monoject® syringe group. Mean PI-NRS scores significantly differed between the iodine tampon group (3.33; SE, 0.27) and Monoject® syringe group (4.46; SE, 0.27) (F (1, 85) = 8.16, *p* < 0.01), with no interaction effect between time and PI-NRS (*F* (6, 510) = 1.26, *p* = 0.28). Patients in the iodine tampon group reported less postoperative discomfort.

**Conclusions:**

Insertion of an iodine-containing tampon in the postoperative socket reduced the pain and impact on oral health-related quality of life during the first postoperative week and positively influenced postoperative sequelae.

## Introduction

Surgical removal of an impacted lower third molar violates the integrity of soft tissues and bone, resulting in postoperative pain, swelling, and trismus and thus negatively impacts quality of life (QoL) [1–5]. A significant reduced QoL as a result of pain has been reported with patients experiencing their greatest pain on the first postoperative day slowly decreasing during the week [3, 4, 6–8]. Postoperative complications like alveolitis and surgical site infection are associated with more and longer-lasting postoperative pain [3].

Many efforts have been studied to prevent or reduce complications after third molar surgery. Antibiotic prophylaxis, chlorhexidine (CHX) mouth rinses, and local corticosteroids have been used to avoid infectious complications and ameliorate pain after mandibular third molar surgery [9–13].

Different studies have reported a beneficial effect of a locally applied gauze drain after the surgical removal of a mandibular third molar on alveolar osteitis, pain, and swelling [14–17]. In a recent cross-over design study of our research group, we found that insertion of an iodine-containing tampon into the extraction alveolus had a positive effect on oral health–related quality of life (OHRQoL), pain, trismus, and several self-care behaviors during the first postoperative week after surgical removal of a mandibular third molar [8].

Recently, a multicenter randomized controlled trial analyzing 333 surgically removed mandibular third molars in 280 patients demonstrated that rinsing out the surgical wound with a Monoject® syringe significantly reduced alveolar osteitis and pain [18].

In the present randomized design, we hypothesized that we would find the same positive effects in patients who received an iodine-containing alveolar tampon on the oral health–related quality of life and pain scores, as well as improved postoperative self-care and discomfort, compared with patients who rinsed the extraction alveolus with a disposable syringe (Monoject®) after wisdom tooth removal as was propagated in the Ghaeminia study [18].

## Materials and methods

### Study design

This prospective randomized controlled trial (RCT) was conducted between April and October of 2018. It was reviewed and approved by the Institutional Ethics Committee (METC) of the Academic Medical Centre of Amsterdam in the Netherlands.

### Study population

Our study included patients who were referred by their dentist for surgical removal of an impacted mandibular third molar at the Department of Oral and Maxillofacial Surgery of the Amstelland Hospital, Amstelveen, the Netherlands. After clinical examination, a panoramic radiograph was taken of each patient. Then, an independent oral and maxillofacial surgeon decided whether the patient met the inclusion criteria. If the patient met the criteria and gave their signed informed consent to participate, the patient was given the Oral Health Impact Profile-14 (OHIP-14) questionnaire with instructions.

### Inclusion and exclusion criteria

This study included only native Dutch speakers who were referred for surgical removal of one impacted mandibular third molar. Other inclusion criteria were age of ≥ 18 years, American Society of Anesthesiology (ASA) score of 1 (i.e., no systemic diseases or medical conditions), no discernible active pathology associated with the third molars, no acute pericoronitis, and no periodontal disease. Exclusion criteria were allergy to ibuprofen or iodine, smoking habit, presence of systemic disease, history of recent and/or symptomatic peptic ulcer, anti-platelet or anticoagulant therapy, pregnancy or lactating, recent local infection within 15 days prior to surgery, previous radiation therapy to the maxillofacial region, local pathology (e.g., cysts or tumor) associated with the third molars, and lack of consent to the procedure or the study.

### Sample size

For sample size calculations, we performed an a priori power analysis using G*Power 3.1.9.4 [19]. Using an independent-samples *t* test, an alpha of 5%, a beta of 15%, one-tailed testing, and an effect size of 0.6, we determined that we needed a sample size of 41 patients per group.

### Randomization and concealment of allocation

This prospective randomized controlled trial (RCT) comprised two groups: an intervention group, which received a postoperative iodine-containing tampon, and a control group, which was instructed to clean their wound with a Monoject® syringe. Following patient inclusion, participants were randomly assigned to a treatment group using a computer randomization generator. The data from the OHIP-14 questionnaire were collected by a student during the follow-up. The questionnaire results were disclosed to the surgeon after statistical analysis of the data.

### Procedures

All surgical procedures were performed by one oral and maxillofacial surgeon. All patients received local anesthesia (articaine hydrochloride 40 mg with 0.01 mg epinephrine, 1.7 mL Ultracain D-S forte; Sanofi-Aventis, Gouda, the Netherlands) to block the inferior alveolar nerve, following the hospital’s protocol. Additionally, infiltration anesthesia was administered in the buccal fold and distal of the incision in the mandibular ramus region.

A triangular incision flap technique was used for all patients [8, 20]. The first incision started from the distobuccal edge of the adjacent second molar, dropping down at a 45° angle with the gingival margin, into the mandibular vestibule. The second incision started laterally in the mandibular ramus and extended to the middle of the second molar, connecting to the distobuccal edge. The mandibular bone surface was exposed, and bone overlying the crown of the wisdom tooth was removed using a surgical bur. The crown was then split using a high-speed turbine handpiece. The bone removal and tooth splitting were accompanied by copious irrigation using sterile saline (0.9% NaCl). Following full removal of the tooth, the alveolus was inspected, and follicular tissue was removed. The socket was rinsed with 10-mL sterile saline (0.9% NaCl).

In the experimental group, an iodine-soaked tampon of 1 × 2 cm (Opraclean; Lohmann & Rauscher BV, Almere, The Netherlands) was placed into the surgical site. The Opraclean tampon is a 100% cotton gauze impregnated with an iodine ointment. The Opraclean dressing supports wound cleaning by absorbing exudate, cell debris, and bacteria and has an antimicrobial effect. In the control group, nothing was placed into the surgical site. In both groups, the surgical wound was sutured using Vicryl Rapide 3/0 (Undyed Vicryl Rapide; Johnson & Johnson, New Brunswick, NJ). The post-extraction socket was not primarily closed in either group.

### Postoperative instructions

Immediately after surgery, patients were given verbal and written postoperative instructions. Patients in both groups were provided with an ice pack for postoperative cooling. Patients in the control group were given additional instructions about how to use the disposable syringe (Monoject®) to rinse the wound 3–4 times daily with tap water for the next week, starting 48 h after surgery. Patients in the tampon group did not receive a disposable syringe. All patients were instructed to bite on a gauze for 30 min. They were also instructed not to rinse or spit during the first 24 postoperative hours. Ibuprofen (Brufen; Abbot BV, Hoofddorp, The Netherlands), 600 mg 3 times a day, was prescribed. No postoperative antibiotics were given. The day after surgery, patients began using 0.12% aqueous chlorhexidine mouth rinse twice a day for 1 min for 7 days. Patients were instructed to complete the daily OHIP-14 questionnaire at the end of the day (before bedtime), and they were recalled for review after 1 week.

### Follow-up

One week after surgery, patients were seen by another surgeon to assess the wound healing of the surgical site and check for alveolitis and wound infection. The patient’s experience of sensory disorders was assessed using a 2-point discrimination test and static light touch detection test. At this time, the completed OHIP-14 questionnaires were collected.

### Outcome measurements

The primary outcome measurements were OHRQoL measured using the OHIP-14, the presence of pain and the pain intensity, and the presence of postoperative sequelae, such as, trismus, swelling, and chewing problems. The secondary outcome measurements were self-care activities, surgical and anatomical variables, and presence of wound infection and alveolar osteitis (AO) [8].

### OHIP-NL14 questionnaire

The participants completed a version of the OHIP-14 that has been translated into Dutch (OHIP-NL14) and evaluated by Van der Meulen et al. [21]. The OHIP-NL14 shows very good internal consistency and reliability (Cronbach’s alpha = 0.90; intraclass correlation coefficient = 0.80) [22]. The questions from the OHIP questionnaire are answered on a 5-point scale that varies from never (0) to very often (4). The total score of the OHIP-14 ranges from 0 to 56, and the separate domain scores provide information regarding the level at which the consequences of the oral problem occur. A higher score on the OHIP-14 indicates a lower quality of life of the patient.

### Pain intensity

We measured pain intensity using an 11-point pain intensity numerical rating scale (PI-NRS). Patients were asked to enter their pain score, ranging from 0 (no pain) to 10 (worst possible pain), on each day of the first postoperative week. Several studies have provided strong support of the validity and reliability of the PI-NRS for detecting changes in pain intensity [23, 24].

### Self-care and discomfort

Self-care and discomfort were measured daily during the first postoperative week. Patients also recorded their intake of prescribed and over-the-counter (OTC) medications. On postoperative day 1 (POD1), the patient reported the number of hours that they used ice packs to cool their cheek on the side of surgery. Patients were also asked to keep a daily record of the presence of swelling, trismus, pain, or inflammatory complications—giving a response of “yes” or “no” for each.

### Statistical analysis

The sample was characterized using conventional descriptive statistics. The chi^2^-test was used to examine associations between categorical variables. Mean scores of multiple measurements in the same subjects were compared using ANOVA for repeated measures. The mean scores between two repeated measurements were compared using the paired-samples *t* test. For skewed data (number of painkillers), analysis was repeated using the Friedman test and the Wilcoxon signed-rank test. An alpha of 5% was set as the level of significance.

## Results

### Description of subjects

A total of 87 subjects participated in this study, including 52 women and 35 men, with an average age of 26.47 years (SD, 6.36 years). These participants were randomly allocated to the experimental group (iodine tampon) or control group (Monoject® syringe). A chi-square test showed that the distribution of men and women did not significantly differ between the two conditions (Table 1). An independent-samples *t* test revealed that the average age was significantly higher in the experimental group compared with the control group. Correlation analysis (Pearson’s) for the age variable and the seven mean OHIP-14 sum scores (repeated measurements of OHIP-14 over seven postoperative days) did not reveal statistically significant correlations; therefore, age was not included as a covariate in follow-up analyses.

**Table 1:** Characteristics of examined groups

	Iodine tampon (*N* = 44)	Monoject® syringe (*N* = 43)	Difference test
Men	17	18	*χ*^2^ = 0.94, df = 1, *p* = 0.76
Women	27	25	36
Average age (SD)	28.11 (7.27)	24.79 (4.80)	*T* = − 2.51, *df* = 74.73, *p* = 0.014

Table 2 presents a frequency table showing the Pell & Gregory classification for both groups [25]. The Mann-Whitney *U* test was used to analyze differences in impaction grade between the two conditions. The results showed that impaction grade did not significantly differ between the iodine tampon group and the Monoject® syringe group (U = 735.00, z = − 1.91, *p* = 0.56).

**Table 2:** Frequency Pell & Gregory classification of impaction of examined groups

Impaction grade	Iodine tampon (*N* = 44)	Monoject® syringe (*N* = 43)	Total
2a	2	3	8
2b	5	5	10
3a	12	20	32
3b	22	15	37
3c	3	0	3
Total	44	43	87

### OHIP-14: iodine tampon versus Monoject® syringe

To determine whether the mean OHIP-14 sum score changed during the first postoperative week in the iodine tampon group and the Monoject® syringe group, we carried out two separate repeated-measures ANOVA (RMA) comparing the means on each postoperative day (Table 3). The results showed a significant effect of time in the iodine tampon group [F (6, 258) = 61.58, *p* < 0.001, *ηp*^2^ = 0.59], as well as in the Monoject® syringe group [F (6, 252) = 108.99, *p* < 0.001, *ηp*^2^ = 0.72]. For each group, pairwise comparison of the mean OHIP-14 sum scores from the seven postoperative days showed that all measurements declined over time and significantly differed from each other (*p* < 0.001).

**Table 3:** Mean OHIP-14 sum scores in the iodine tampon and Monoject® syringe conditions

Intervention OHIP-14 Mean (SD)	Iodine tampon (*N* = 44)	Monoject® syringe (*N* = 43)	*p* value^a^
Day 1	20.84 (9.11)	27.79 (10.19)	0.001
Day 2	17.16 (11.15)	23.07 (10.08)	0.011
Day 3	12.91 (10.69)	18.84 (10.25)	0.010
Day 4	9.72 (9.09)	15.29 (9.51)	0.006
Day 5	7.71 (7.73)	11.93 (9.13)	0.022
Day 6	5.64 (6.48)	8.66 (8.35)	0.064
Day 7	4.19 (5.93)	5.50 (6.20)	0.319

^a^*p* value from independent-samples *t*-test for differences in mean OHIP-14 sum scores for each of the 7 post-operative days between the iodine tampon and Monoject® syringe conditions

**Fig. 1:**
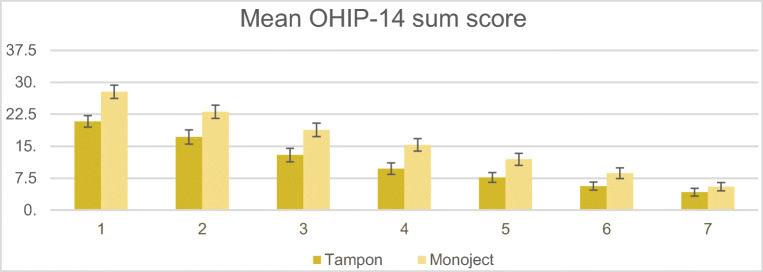
OHIP-14 sum scores on the seven postoperative days for both conditions

We next assessed the extent to which the mean OHIP-14 sum scores differed between the two interventions across the multiple measurements, by performing a repeated-measures ANOVA between-subjects factor. The results indicated that there was a statistically significant interaction effect between the factor of time and each intervention (iodine tampon and Monoject® syringe) [*F* (*df* = 6, 510) = 3.27, *p* = 0.004, *ηp*^*2*^ = 0.037]. This meant that the changes in the mean OHIP-14 score over time differed between the two conditions (Fig. 1).

To investigate the source of the significant interaction effect between the two groups, first, we calculated a mean difference score (i.e., change over time) between the first and second postoperative day, between the second and third postoperative day, etc. Next, we compared the two patient groups with regard to the mean changes for each calculated difference score. Table 4 presents the comparison of differences between the mean OHIP-14 sum scores for the two conditions. Independent-samples *t* tests revealed that the difference Δ7–6 was statistically significant (*p* = 0.048). The independent-samples *t* test enabled examination of whether the conditions differed in the mean OHIP-14 sum scores for the 7 postoperative days (Table 3). We found that the mean OHIP-14 sum scores were significantly lower in the iodine tampon group than in the Monoject® syringe group, except on postoperative days 6 and 7.

**Table 4:** Independent-sample *t* tests of differences in mean delta OHIP-14 between the iodine tampon and Monoject® syringe conditions

Intervention Differences between POD	Iodine tampon (*N* = 44) Mean (SD)	Monoject® syringe (*N* = 43) Mean (SD)	*p* value
Δ 2–1	− 3.68 (9.32)	− 4.72 (5.21)	0.52
Δ 3–2	− 4.25 (4.24)	− 4.23 (4.50)	0.99
Δ 4–3	− 3.19 (4.84)	− 3.55 (4.94)	0.74
Δ 5–4	− 2.01 (3.96)	− 3.36 (5.02)	0.17
Δ 6–5	− 2.06 (3.22)	− 3.27 (4.58)	0.16
Δ 7–6	− 1.46 (3.69)	− 3.16 (4.24)	0.048*

*p < 0.05

### PI-NRS: iodine tampon versus Monoject® syringe

To assess the use of an iodine tampon compared with the Monoject® syringe in terms of the measured pain intensity score during the first postoperative week, we carried out two separate RMA and independent-samples *t* tests, comparing the PI-NRS scores for both groups on each postoperative day. RMA analysis revealed a statistically significant effect of time in the iodine tampon group [F (6, 258) = 46.48, *p* < 0.001, *ηp*^2^ = 0.52] as well as in the Monoject® syringe group [F (6, 252) = 57.64, *p* < 0.001, *ηp*^2^ = 0.58]. A pairwise comparison of the PI-NRS score over seven postoperative days in the iodine tampon group revealed that all measurements declined over time and significantly differed from each other, except between postoperative days 1 and 2 (*p* = 0.065). Pairwise comparison of the mean PI-NRS score over seven postoperative days in the Monoject® syringe group showed that the mean scores declined over time, with significant differences between all days (*p* < 0.05).

We additionally carried out a repeated-measures ANOVA between-subjects factor analysis to evaluate differences in the PI-NRS between the two conditions. The results revealed that the mean PI-NRS significantly differed between the iodine tampon group (3.33; SE, 0.27) and Monoject® syringe group (4.46; SE, 0.27) [F (1, 85) = 8.16, *p* < 0.01], and we found no interaction effect between time and condition on the PI-NRS [*F* (6, 510) = 1.26, *p* = 0.28].

To determine the effect of the iodine tampon compared with use of the Monoject® syringe, we performed independent-samples *t* test analysis on PI-NRS scores for the seven postoperative days. Table 5 shows the differences in mean PI-NRS scores between the iodine tampon and Monoject® syringe conditions on each postoperative day. The results showed that the mean PI-NRS scores in the iodine tampon group were significantly lower from postoperative day 1 up to and including postoperative day 4. The two groups did not significantly differ on the subsequent postoperative days.

**Table 5:** Mean PI-NRS scores in the iodine tampon and Monoject® syringe conditions

Intervention PI- NRS Avg. (SD)	Iodine tampon (*N* = 44)	Monoject® syringe (*N* = 43)	*p* value^a^
Day 1	5.22 (2.32)	6.61 (1.70)	0.002
Day 2	4.74 (2.50)	5.84 (1.94)	0.025
Day 3	3.94 (2.58)	5.40 (1.78)	0.003
Day 4	3.16 (2.41)	4.69 (1.91)	0.002
Day 5	2.73 (2.41)	3.56 (2.14)	0.093
Day 6	2.14 (2.26)	3.02 (2.33)	0.075
Day 7	1.41 (2.08)	2.12 (2.10)	0.116

^a^*p* value from independent *t*-test for differences in mean PI-NRS scores for 7 post-operative days between the iodine tampon and Monoject® syringe conditions

### Discomfort and self-care

Table 6 presents the results concerning the variables on self-care and discomfort, which clearly demonstrated a superior effect of iodine-containing tampons during the first postoperative week after extraction. Notably, on postoperative day 4, 57% of patients in the intervention group used the prescribed medication, compared with 84% in the control group. Similar results were found with regard to the presence of limited mouth opening (trismus), chewing problems, swollen cheek, and pain. Additionally, “no discomfort at all” was reported on postoperative day 4 by 2% of patients in the intervention group compared with 0% of the control group, and on postoperative day 7 by 45% of the intervention group compared with 30% of the control group. The two groups reported a similar number of hours that they cooled their cheek with an ice pack on the first day after surgery, and an independent-samples *t* test showed no significant difference groups in the mean number of cooling hours [t (85) = .97, *p* = 0.33; mean, 5.3 h; range, 0–12 h].

**Table 6:** Percentage of patients who answered “yes” on the self-care and discomfort questions

Question (yes)	Intervention	Day 1	Day 2	Day 3	Day 4	Day 5	Day 6	Day 7
Did you use the prescribed medication?	Monoject	100	93	91	84	65	49	40
	Tampon	98	89	86	57	48	34	25
Did you cool with an ice package?	Monoject	100	51	23	14	14	2	0
	Tampon	100	45	25	14	9	5	5
Did you use additional medication other than that prescribed?	Monoject	26	16	19	14	19	14	7
	Tampon	18	14	16	18	11	9	9
Did you follow the same routine as always?	Monoject	0	2	5	12	26	47	60
	Tampon	2	11	20	32	50	64	73
Did you experience limited mouth opening?	Monoject	100	98	93	79	70	42	37
	Tampon	98	93	86	73	57	39	23
Did you experience reduced chewing ability?	Monoject	93	91	91	81	72	42	30
	Tampon	91	80	70	73	57	45	30
Did you experience a swollen cheek?	Monoject	93	95	95	86	63	37	26
	Tampon	82	93	93	77	43	23	11
Did you experience pain as a result of surgery?	Monoject	98	93	88	79	65	63	47
	Tampon	86	84	80	64	59	45	25
Did you experience any discomfort?	Monoject	0	0	0	0	0	12	30
	Tampon	0	0	0	2	16	25	45

### Duration of surgery

The mean duration of surgery in the control group was 11.07 min (SD, 1.10 min). The mean surgery duration in the intervention group was significantly longer: 12.18 min (SD, 2.64 min).

### Postoperative complications

There were no cases of postoperative infection or AO. Temporary hypoesthesia occurred in two cases (0.5%) in the control group after surgical removal of the third molar. Full recovery of sensibility was observed in both patients after 6 months.

## Discussion

In the present study, we aimed to assess how the use of an alveolar iodine-containing tampon affected postoperative oral health–related quality (OHRQoL) following third mandibular molar surgery. In accordance with previous findings, surgical removal of the impacted mandibular third molar significantly affected OHRQoL during the first postoperative days [1–8]. Postoperative sequelae, such as pain, trismus, swelling, and chewing problems, commonly arise after tissue injury. Numerous researchers have studied the effects of various preoperative, intraoperative, and postoperative intervention strategies to avoid or decrease the degree of discomfort due to inflammation induced by tissue injury during the surgical removal of mandibular third molars [26–34]. Here, we demonstrated that the application of an iodine-containing tampon reduced the amount of perceived postoperative discomfort, and thus improved the OHRQoL.

We used the OHIP-14 questionnaire to evaluate the effects of iodine-containing tampons on the physical, social, psychological, and functional aspects of daily life. Daily measurement of the mean OHIP-14 sum scores in the iodine tampon group revealed that the scores significantly decreased each day from the first postoperative day to the seventh. In the Monoject® syringe group, the mean OHIP-14 sum scores for postoperative days 1 and 2 were similar to the values in a 2012 study by Kieffer et al. [20]. However, on postoperative day 3, the mean OHIP-14 sum score was lower in the Monoject® syringe group in our study compared with Kieffer’s study. This indicated that postoperative irrigation of the extraction socket was beneficial to decrease the amount of discomfort. Ghaeminia et al. previously reported benefits of the use of a Monoject® syringe after mandibular third molar surgery [18]. In the present study, the postoperative extraction sockets were not primarily closed, and thus remained a vulnerable site for debris accumulation.

The other major primary outcome measure in our study was the effect of alveolar iodine–containing tampons on postoperative pain intensity. For pain relief, the patients were prescribed ibuprofen 600 mg, commencing immediately after the surgery. Bailey and colleagues proposed that NSAIDS, such as ibuprofen, should be considered the first choice of pain relief medication [35]. In addition to the prescribed ibuprofen 600 mg, the patients in our study also reported the type and dosage of any other over-the-counter (OTC) medications used. Most patients reported the intake of paracetamol 1000 mg in combination with their prescribed medication. This combination is reportedly beneficial for pain relief after third molar surgery.

Both study groups reported pain on postoperative day 1. The Monoject® syringe group exhibited a slightly higher pain score on the 1st postoperative day, and the two study groups significantly differed in pain perception on the following days. Additionally, the Monoject® syringe group had a higher percentage of patients with intake of prescribed medications on all assessed postoperative days. On the 4th postoperative day, less than 60% of patients in the iodine tampon group were taking prescribed medications, compared with over 80% of the patients in the Monoject® syringe group. The two groups also showed differences in other clinical parameters commonly induced as the result of inflammatory responses, such as swelling, trismus, and chewing problems. The differences in these clinical parameters appeared to be higher after the 3rd postoperative day. From these results, it was obvious that the iodine-containing tampon group suffered less postoperative inconvenience.

Several factors have been identified as risk factors for the severity of postoperative sequelae [31, 36], including patient’s age, gender, anatomical and surgical variables (e.g., degree of impaction), wound closure techniques, operator experience, and the procedure duration. In the present study, the two groups did not significantly differ in the distribution of men and women, but a *t* test for independent observations revealed a significant between-group difference in age. However, Pearson’s correlation analysis did not reveal a statistically significant correlation between age and mean OHIP-14 sum scores. This finding is in accordance with results presented by Benediktsdóttir et al. [37]. Moreover, the independent *t* test showed no statistically significant correlation between gender and mean OHIP-14 sum scores. All procedures were performed by one specialized oral and maxillofacial surgeon; therefore, operator experience did not influence the results and could be eliminated as a variable adversely effecting OHRQoL [7, 38–40]. The mean operating duration was significantly lower for the control group (11.07 min) compared with the intervention group (12.18 min), but the difference was clinically irrelevant.

Many prior studies have evaluated how different wound closure techniques influence the degree of discomfort after the surgical removal of third molars. There remains considerable controversy, with some studies suggesting that an open wound may be beneficial [41–44], while others found that primary closure of the wound is more convenient [45]. In both groups of our present study, the postoperative extraction sockets were left open for healing by means of secondary intention. The patients in the Monoject® syringe group were instructed to irrigate the post-extraction socket with tap water at 48 h postoperatively. For patients in the experimental group, an iodine-containing tampon was placed in the extraction socket after third molar extraction. Wound healing by secondary intention and administration of an alveolar iodine-containing tampon in the postoperative extraction socket is a form of surgical drainage.

Over the past three decades, multiple studies have examined the administration of various foreign agents in a post-extraction socket [14–17] and have evaluated how these agents impact the degrees of pain, swelling, trismus, and chewing problems. Additionally, several prior studies have evaluated the effects of surgical drainage on wound healing, postoperative sequelae, and pain. Hollander et al. observed reduced postoperative pain and swelling when using a bismuth iodoform paraffin paste–impregnated (BIPP) ribbon gauze dressing with partial closure, compared with a primary closure technique [14]. Similarly, Egbor et al. reported reduced postoperative swelling and trismus in patients treated with a Whitehead’s Varnish dressing in the socket, compared with primary closure [15]; however, the measured pain score did not significantly differ between these study populations. Notably, all patients received oral administration of 500 mg amoxicillin and 200 mg metronidazole for 5 days postoperatively, and thus, it is unclear whether the positive effects can be fully attributed to the dressing intervention. Consistent findings were also described by Chukwuneke et al. [46] and Chaudhary et al. [47].

Liu et al. [17] performed a systematic review of ten randomized controlled trials to evaluate the effectiveness of surgical drainage after mandibular third molar surgery. They concluded that surgical drainage has a positive effect on postoperative sequelae, resulting in less swelling and trismus during the early and late stages, and significantly less pain during the early stage. They also evaluated three types of drainage methods and concluded that the tube drain group showed better results than the rubber drain and gauze drain groups, due to a stronger drainage effect. Akota et al. [16] assessed the post-surgical effects of locally applied gauze drain impregnated with chlortetracycline ointment and concluded that the impregnated drain effectively reduced alveolar osteitis. However, they did not find any beneficial effects on postoperative pain, swelling, or trismus. Rakprasitkul et al. [48] compared primary closure with placement of a tube drain after surgery and found that surgical drainage did not influence pain but had a significant positive effect on postoperative swelling and trismus, which is in agreement with the finding of Egbor et al. [15].

Benediktsdóttir et al. reported that the use of an ice pack to cool the masseteric region, starting immediately after surgery, resulted in significantly reduced swelling and trismus (*p* < 0.05) on postoperative days 1, 2, and 7 [37]. However, Van der Westhuijzen et al. [49] and Zandi et al. [50] did not find any significant difference in postoperative sequelae with the application of an ice pack after third mandibular surgery in their studies. In our present study, immediately after the operation, patients in both groups were given an ice pack and instructed to apply it to the cheek on the side of intervention in 10-min intervals. Although both groups used ice packs, the two groups in our study exhibited significantly different degrees of swelling, based on the overall mean OHIP-14 sum scores measured on the first 3 days postoperatively. Notably, Benediktsdóttir et al. reported that level of impaction was correlated with postoperative pain [37]. In our present study, the Mann-Whitney *U* test showed that no significant differences in impaction grade between the iodine tampon group and the Monoject® syringe group (*U* = 735.00, *z* = − 1.91, *p* = 0.56). However, the iodine tampon group included more patients with a higher impaction grade compared with the Monoject® syringe group. Thus, with all other things being equal, the iodine tampon group was at a greater risk for postoperative pain. Considering that patients in the iodine tampon group perceived less postoperative sequelae, it is likely that the effect of an alveolar iodine tampon on OHRQoL would have been even greater than in our present results if both groups had been equal.

Chlorhexidine (CHX) has an antimicrobial effect that can last up to 24 h. Several studies have evaluated the effect of a CHX rinse on the incidence of AO [12, 13]. Rinsing preoperatively and up to 7 days postoperatively with CHX 0.12% significantly reduces the incidence of AO. On the other hand, a single preoperative rinse with CHX was not associated with a significant reduction in AO incidence [51]. Adverse side effects, such as tooth discoloration and alteration in taste, have been reported with prolonged use of CHX [52, 53]; therefore, it is advised that CHX use should be limited to a short period. In our present study, the incidence of surgical site infection (SSI) and AO was 0%. These results were positive compared with the prevalence rates reported in other studies, which vary between 1 and 30% and between 3.9 and 29.6% respectively [54, 55].

Despite much effort to objectify our present results, there are several limitations that must be considered when interpreting the results. All third molars removed in the present study were asymptomatic and without pathology; therefore, no statements can be made about the effectiveness of placing an iodine-containing tampon in the post-extraction socket in cases of active pathology. Additionally, this study only measured the effects of the iodine-containing tampon after surgical removal of the mandibular third molars; therefore, our results cannot be extrapolated to other extraction sites in the tooth arch. Another limitation is that there is a lack of data regarding the correct usage of the Monoject® syringe by the patients. Failing to correctly rinse the postoperative extraction socket after surgery may lead to food impaction, infection, and delayed healing time. Ghaeminia et al. reported that 42% of the patients were unable to irrigate the postoperative extraction socket, despite having received instructions [18]. This issue may have resulted in more postoperative sequelae for the control group, and thus adversely affected patients’ QoL. Finally, the data regarding the postoperative days were filled in by the patients themselves. Although self-assessment or self-reporting is a preferred method for data acquisition, the data are subjective, and the assessment of self-reported data is not immune to potential bias [56]. A recall on postoperative day 3 or 4 would have been helpful for objective assessment of the clinical parameters.

## Conclusion

The results of our present study indicated that the administration of an alveolar iodine–containing tampon in the postoperative extraction socket, after removal of an impacted mandibular third molar, resulted in improved OHIP-14 and PI-NRS scores. The use of an iodine tampon also had positive effects on postoperative sequelae, and thereby resulted in less postoperative inconvenience and discomfort following the surgical removal of an impacted mandibular third molar.

## References

[1] Duarte-Rodrigues L, Miranda EFP, de Paiva HN, Falci SGM, Galvão EL, Souza TO (2018). Third molar removal and its impact on quality of life: systematic review and meta-analysis. Qual Life Res.

[2] McGrath C, Comfort MB, Lo ECM, Luo Y (2003). Changes in life quality following third molar surgery–the immediate postoperative period. Br Dent J.

[3] van Wijk AJ, Kieffer JM, Lindeboom JA (2009). Effect of third molar surgery on oral health-related quality of life in the first postoperative week using Dutch version of Oral Health Impact Profile-14. J Oral Maxillofac Surg.

[4] Deepti C, Rehan HS, Mehra P (2009). Changes in quality of life after surgical removal of impacted mandibular third molar teeth. J Maxillofac Oral Surg.

[5] Ibikunle AA, Adeyemo WL (2017). Oral health-related quality of life following third molar surgery in an African population. Contemp Clin Dent.

[6] Conrad SM, Blakey GH, Shugars DA, Marciani RD, Phillips C, White RP (1999). Patients’ perception of recovery after third molar surgery. J Oral Maxillofac Surg.

[7] Lago-Méndez L, Diniz-Freitas M, Senra-Rivera C, Gude-Sampedro F, Gándara Rey JM, García-García A (2007). Relationships between surgical difficulty and postoperative pain in lower third molar extractions. J Oral Maxillofac Surg.

[8] Tuk JG, Lindeboom JA, Sana F, van Wijk AJ, Milstein DMJ (2019). Alveolar iodine tampon packing reduces postoperative morbidity after third molar surgery. J Oral Maxillofac Surg.

[9] Morrow AJ, Dodson TB, Gonzalez ML, Chuang SK, Lang MS (2018). Do postoperative antibiotics decrease the frequency of inflammatory complications following third molar removal?. J Oral Maxillofac Surg.

[10] Ramos E, Santamaría J, Santamaría G, Barbier L, Arteagoitia I (2016). Do systemic antibiotics prevent dry socket and infection after third molar extraction? A systematic review and meta-analysis. Oral Surg Oral Med Oral Pathol Oral Radiol.

[11] Graziani F, D'Aiuto F, Arduino PG, Tonelli M, Gabriele M (2006). Perioperative dexamethasone reduces post-surgical sequelae of wisdom tooth removal. A split-mouth randomized double-masked clinical trial. Int J Oral Maxillofac Surg.

[12] Larsen PE (1991). The effect of a chlorhexidine rinse on the incidence of alveolar osteitis following the surgical removal of impacted mandibular third molars. J Oral Maxillofac Surg.

[13] Caso A, Hung LK, Beirne OR (2005). Prevention of alveolar osteitis with chlorhexidine: a meta-analytic review. Oral Surg Oral Med Oral Pathol Oral Radiol Endod.

[14] Holland CS, Hindle MO (1984). The influence of closure or dressing of third molar sockets on post-operative swelling and pain. Br J Oral Maxillofac Surg.

[15] Egbor PE, Saheeb BD (2014). A prospective randomized clinical study of the influence of primary closure or dressing on postoperative morbidity after mandibular third molar surgery. Niger J Surg.

[16] Akota I, Alvsaker B, Bjørnland T (1998). The effect of locally applied gauze drain impregnated with chlortetracycline ointment in mandibular third-molar surgery. Acta Odontol Scand.

[17] Liu S, You Z, Ma C, Wang Y, Zhao H (2018). Effectiveness of drainage in mandibular third molar surgery: a systematic review and meta-analysis. J Oral Maxillofac Surg.

[18] Ghaeminia H, Hoppenreijs TJ, Xi T, Fennis JP, Maal TJ, Bergé SJ, Meijer GJ (2017). Postoperative socket irrigation with drinking tap water reduces the risk of inflammatory complications following surgical removal of third molars: a multicenter randomized trial. Clin Oral Investig.

[19] Faul F, Erdfelder E, Lang AG, Buchner A (2007). G*Power 3: a flexible statistical power analysis program for the social, behavioral, and biomedical sciences. Behav Res Methods.

[20] Kieffer JM, van Wijk AJ, Ho JP, Lindeboom JA (2012). The internal responsiveness of the oral health impact Profile-14 to detect differences in clinical parameters related to surgical third molar removal. Qual Life Res.

[21] van der Meulen MJ, John MT, Naeije M, Lobbezoo F (2008). The Dutch version of the Oral health impact profile (OHIP-NL): translation, reliability and construct validity. BMC Oral Health.

[22] Isik K, Unsal A, Kalayci A, Durmus E (2011). Comparison of three pain scales after impacted third molar surgery. Oral Surg Oral Med Oral Pathol Oral Radiol Endod.

[23] Lara-Munoz C, De Leon SP, Feinstein AR, Puente A, Wells CK (2004). Comparison of three rating scales for measuring subjective phenomena in clinical research. I. Use of experimentally controlled auditory stimuli. Arch Med Res.

[24] Williamson A, Hoggart B (2005). Pain: a review of three commonly used pain rating scales. J Clin Nurs.

[25] Pell GJ, Gregory BT (1933). Impacted mandibular third molars: classification and modified techniques for removal. Dent Dig.

[26] Arteagoitia I, Ramos E, Santamaria G, Barbier L, Alvarez J, Santamaria J (2015). Amoxicillin/clavulanic acid 2000/125 to prevent complications due to infection following completely bone-impacted lower third molar removal: a clinical trial. Oral Surg Oral Med Oral Pathol Oral Radiol.

[27] Martín-Ares M, Barona-Dorado C, Martínez-Rodríguez N, Cortés-Bretón-Brinkmann J, Sanz-Alonso J, Martínez-González JM (2017). Does the postoperative administration of antibiotics reduce the symptoms of lower third molar removal? A randomized double-blind clinical study. J Clin Exp Dent.

[28] Bloomer CR (2000). Alveolar osteitis prevention by immediate placement of medicated packing. Oral Surg Oral Med Oral Pathol Oral Radiol Endod.

[29] Ibikunle AA, Adeyemo WL, Ladeinde AL (2016). Oral health-related quality of life following third molar surgery with either oral administration or submucosal injection of prednisolone. Oral Maxillofac Surg.

[30] Alexander RE, Throndson RR (2000). A review of perioperative corticosteroid use in dentoalveolar surgery. Oral Surg Oral Med Oral Pathol Oral Radiol Endod.

[31] Herrera-Briones FJ, Prados Sánchez EP, Reyes Botella CR, Vallecillo Capilla MV (2013) Update on the use of corticosteroids in third molar surgery: systematic review of the literature. Oral Surg Oral Med Oral Pathol Oral Radiol 116:e342–e351 10.1016/j.oooo.2012.02.027 22902498

[32] Cheung HY, Wong MMK, Cheung SH, Liang LY, Lam YW, Chiu SK (2012). Differential actions of chlorhexidine on the cell wall of Bacillus subtilis and Escherichia coli. PLoS One.

[33] Hita-Iglesias P, Torres-Lagares D, Flores-Ruiz R, Magallanes-Abad N, Basallote-Gonzalez M, Gutierrez-Perez JL (2008). Effectiveness of chlorhexidine gel versus chlorhexidine rinse in reducing alveolar osteitis in mandibular third molar surgery. J Oral Maxillofac Surg.

[34] Jadhao VA, Rao A, Hande P, Mahajani M, Raktade PP, Gedam R, Tekale PD (2018). The efficiency of three irrigating solutions after surgical removal of impacted mandibular third molars: a cross-sectional study. J Contemp Dent Pract.

[35] Bailey E, Worthington HV, van Wijk AJ, Yates JM, Coulthard P, Afzal Z (2013). Ibuprofen and/or paracetamol (acetaminophen) for pain relief after surgical removal of lower wisdom teeth. Cochrane Database Syst Rev.

[36] Chuang SK, Perrott DH, Susarla SM, Dodson TB (2007). Age as a risk factor for third molar surgery complications. J Oral Maxillofac Surg.

[37] Benediktsdóttir IS, Wenzel A, Petersen JK, Hintze H (2004). Mandibular third molar removal: risk indicators for extended operation time, postoperative pain, and complications. Oral Surg Oral Med Oral Pathol Oral Radiol Endod.

[38] Tenglikar P, Munnangi A, Mangalgi A, Uddin SF, Mathpathi S, Shah K (2017). An assessment of factors influencing the difficulty in third molar surgery. Ann Maxillofac Surg.

[39] Jerjes W, El-Maaytah M, Swinson B, Banu B, Upile T, D’Sa S, Al-Khawalde M, Chaib B, Hopper C (2006) Experience versus complication rate in third molar surgery. Head Face Med 25:2–14 10.1186/1746-160X-2-14 PMC1481631 16725024

[40] Vlcek D, Razavi A, Kuttenberger JJ (2015). Wound management and the use of mouth rinse in mandibular third molar surgery. Swiss Dent J.

[41] de Brabander EC, Cattaneo G (1988). The effect of surgical drain together with a secondary closure technique on postoperative trismus, swelling and pain after mandibular third molar surgery. Int J Oral Maxillofac Surg.

[42] Pasqualini D, Cocero N, Castella A, Mela L, Bracco P (2005). Primary and secondary closure of the surgical wound after removal of impacted mandibular third molars: a comparative study. Int J Oral Maxillofac Surg.

[43] Pachipulusu PK, Manjula S (2018). Comparative study of primary and secondary closure of the surgical wound after removal of impacted mandibular third molars. Oral Maxillofac Surg.

[44] Danda AK, Krishna Tatiparthi M, Narayanan V, Siddareddi A (2010). Influence of primary and secondary closure of surgical wound after impacted mandibular third molar removal on postoperative pain and swelling - a comparative and split mouth study. J Oral Maxillofac Surg.

[45] Dubois DD, Pizer ME, Chinnis RJ (1982). Comparison of primary and secondary closure techniques after removal of impacted mandibular third molars. J Oral Maxillofac Surg.

[46] Chukwuneke FN, Oji C, Saheeb DB (2008). A comparative study of the effect of using a rubber drain on postoperative discomfort following lower third molar surgery. Int J Oral Maxillofac Surg.

[47] Chaudhary M, Singh M, Singh S, Singh SP, Kaur G (2012). Primary and secondary closure technique following removal of impacted mandibular third molars: a comparative study. Natl J Maxillofac Surg.

[48] Rakprasitkul S, Pairuchvej V (1997). Mandibular third molar surgery with primary closure and tube drain. Int J Oral Maxillofac Surg.

[49] van der Westhuijzen AJ, Becker PJ, Morkel J, Roelse JA (2005). A randomized observer blind comparison of bilateral facial ice pack therapy with no ice therapy following third molar surgery. Int J Oral Maxillofac Surg.

[50] Zandi M, Amini P, Keshavarz A (2016). Effectiveness of cold therapy in reducing pain, trismus, and oedema after impacted mandibular third molar surgery: a randomized, self-controlled, observer-blind, split-mouth clinical trial. Int J Oral Maxillofac Surg.

[51] Cho H, David MC, Lynham AJ, Hsu E (2018). Effectiveness of irrigation with chlorhexidine after removal of mandibular third molars: a randomised controlled trial. Br J Oral Maxillofac Surg.

[52] Gagari E, Kabani S (1995). Adverse effects of mouthwash use: a review. Oral Surg Oral Med, Oral Pathol, Oral Radiol Endod.

[53] Guerra F, Pasqualotto D, Rinaldo F, Mazur M, Corridore D, Nofron I, Nardi GM (2019). Therapeutic efficacy of chlorhexidine-based mouthwashes and its adverse events: performance-related evaluation of mouthwashes added with anti-discoloration system and cetylpyridinium chloride. Int J Dent Hyg.

[54] Hermesch CB, Hilton TJ, Biesbrock AR, Baker RA, Cain-Hamlin J, McClanahan SF, Gerlach RW (1998). Perioperative use of 0.12% chlorhexidine gluconate for the prevention of alveolar osteitis: efficacy and risk factor analysis. Oral Surg Oral Med Oral Pathol Oral Radiol Endod.

[55] Upadhyaya C, Humagain H (2010). Prevalence of dry socket following extraction of permanent teeth at Kathmandu University Teaching Hospital (KUTH), Dhulikhel, Kavre, Nepal: a study. Kathmandu Univ Med J (KUMJ).

[56] Donaldson SI, Grant-Vallone EJ (2002). Understanding self-report bias in organizational behavior research. J Bus Psychol.

